# Supplementary material for: The CPLANE protein Intu protects kidneys from ischemia-reperfusion injury by targeting STAT1 for degradation
Source: Nat Commun. 2018 Mar 26;9:1234. doi: 10.1038/s41467-018-03628-8 (PMC5964315; doi:10.1038/s41467-018-03628-8)
Supplement: Supplementary file 1 — Supplementary Information(PDF 2744 kb) [file 41467_2018_3628_MOESM1_ESM.pdf]

The CPLANE protein Intu protects kidneys from ischemia-reperfusion injury by targeting STAT1 for degradation

Wang *et al*.

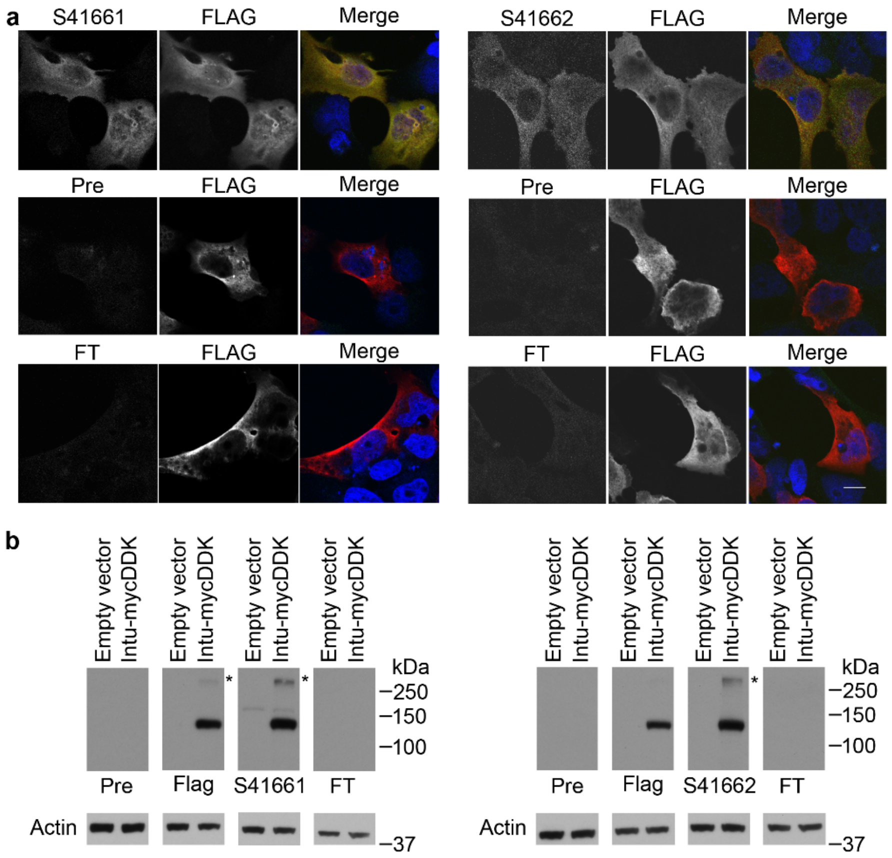

Supplementary Figure 1 Generation and characterization of Intu antibody. To test the specificity and sensitivity of Intu antibodies, Intu construct tagged by c-Myc and DDK (FLAG) at the N-terminus, was transfected into 293FT cells. With antibodies to FLAG and Intu (S41661 and S41662) from two different immunized rabbits respectively, we have performed immunostaining (**a**) and immunoblot (**b**). In contrast to the preimmune (Pre) and flow-through (FT) sera, affinity purified Intu antibodies demonstrated clear and strong signals, compared to the empty vector-transfected cells. * indicates a likely dimer of Intu. Cell nuclei were stained with DAPI. Scale bar, 10 μm.

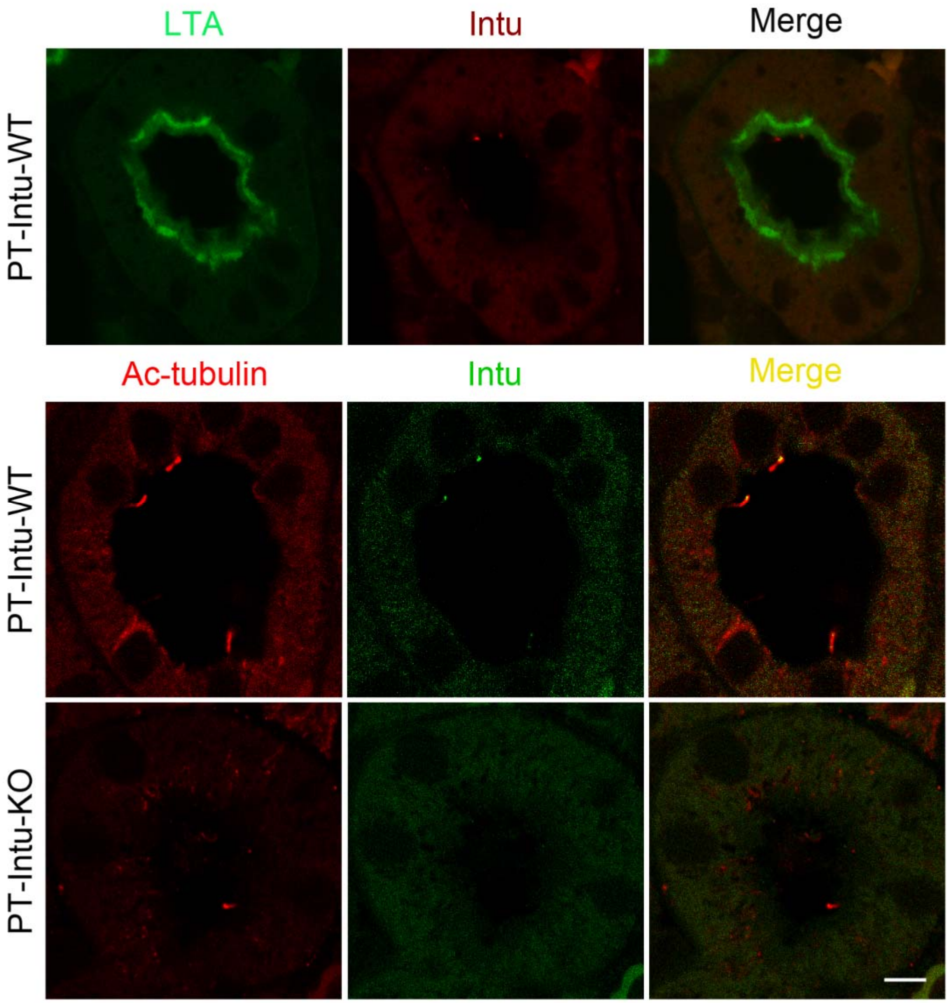

Supplementary Figure 2 Intu ablation in *Intu* knockout cells. To confirm the knockout of Intu in renal tubular cells, we performed immunofluorescence staining with mouse kidney sections. Intu was detected specifically at the base of primary cilia labelled with Ac-tubulin in proximal tubules marked with fluorescein LTA. In *Intu* KO cells, Intu was not detected. Scale bar, 5 μm.

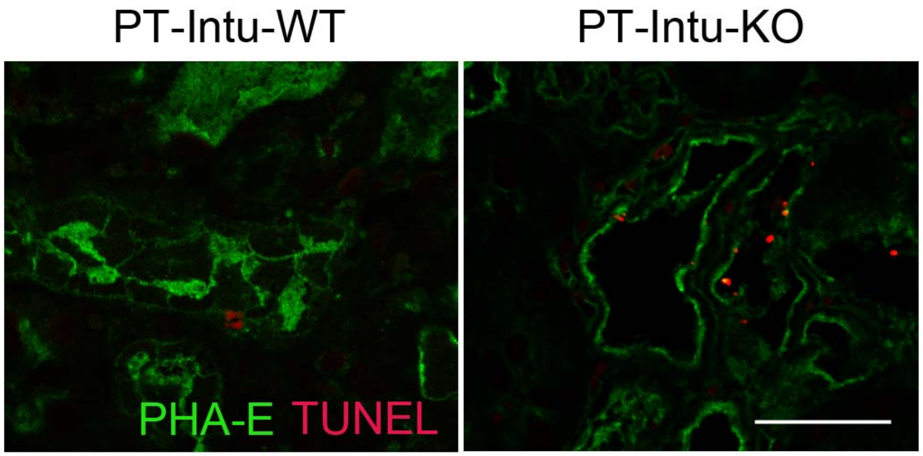

Supplementary Figure 3 TUNEL assay on *Intu* KO kidney sections after renal I/R injury. More death cells (red) were observed in PT-Intu-KO renal proximal tubules, labelled with fluorescein PHA-E, than in PT-Intu-WT mice. Scale bar, 50 μm.

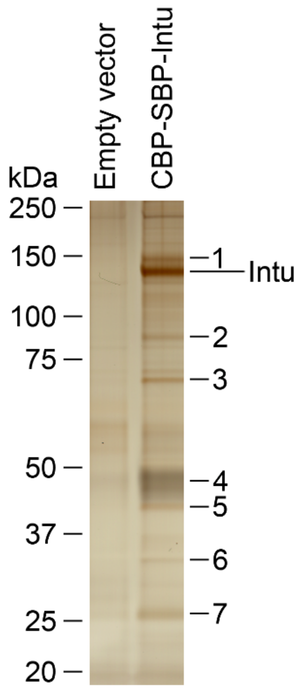

Supplementary Figure 4 Silver staining on SDS-PAGE gel. Overexpressed Intu in BUMPT cells was pulled down with streptavidin and calmodulin resins and run in 4-12% SDS-PAGE gel, followed by the silver staining. Seven bands were removed for the mass spectrometry analysis. Please note, the strongest band pulled down is Intu proteins.

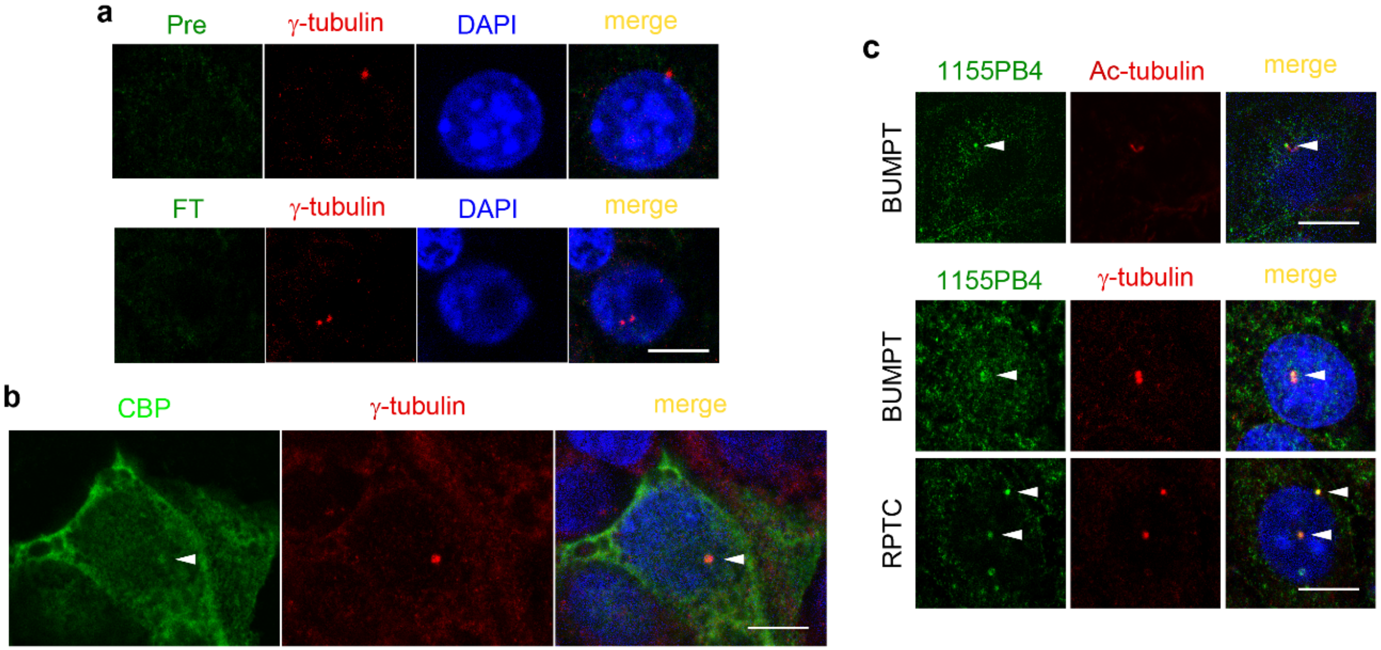

Supplementary Figure 5 (**a**) Double staining with preimmune (Pre) or flow-throw (FT) sera for antibody S41662 and γ–tubulin in BUMPT cells. No signal was detected with Pre and FT sera. (**b**) Recombinant Intu construct (CBP-SBP-Intu) was transfected into 293FT cells and co-stained with CBP-tag and γ-tubulin antibodies. Exogenous Intu trafficked to the centriole area. (**c**) Double immunostaining of BUMPT and RPTC cells with 1155PB4 and Ac-tubulin/γ-tubulin antibodies. Cell nuclei were stained by DAPI. Scale bar, 10 μm.

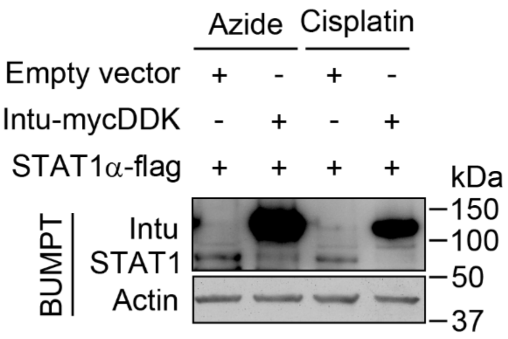

Supplementary Figure 6 Intu overexpression suppressed the expression level of STAT1 in BUMPT cells upon azide and cisplatin treatment respectively.

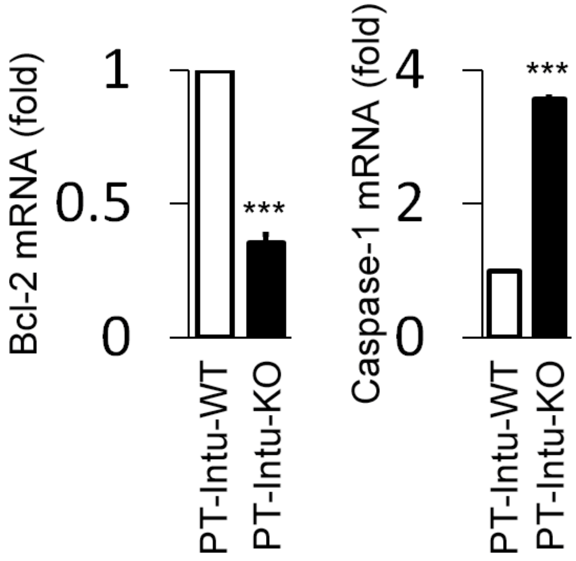

Supplementary Figure 7 Quantitative real-time PCR analysis for Bcl-2 and Caspase-1 in mice. To explore the transcriptional regulation of Bcl-2 and Caspase-1, we took advantage of qRT PCR to measure the transcript levels of these two genes. Bcl-2 transcript level was down-regulated while Caspase-1 upregulated in PT-Intu-KO mice, in comparison to PT-Intu-WT mice. Quantitative data are mean ± s.d. (error bar). n=3. Paired *t* test was used. *p* *** < 0.001.

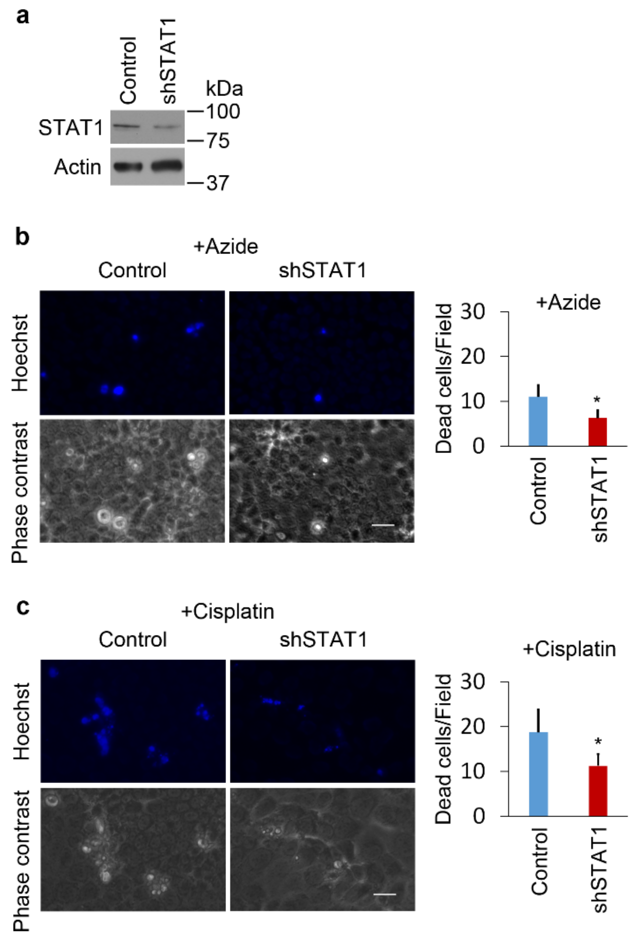

Supplementary Figure 8 To determine the effect of STAT1 reduction on cell death, we knocked down STAT1 in BUMPT cells. (**a**) STAT1 knockdown was confirmed by immunoblot. (**b, c**) After exposure to azide for 3 h or cisplatin for 24 h, STAT1 knockdown cells displayed resistance to cell death. Quantitative data are mean ± s.d. (error bar). n=4. Paired *t* test was used. *p* * < 0.05. Cell nuclei were stained with Hoechst 33342. Scale bar, 20 μm.

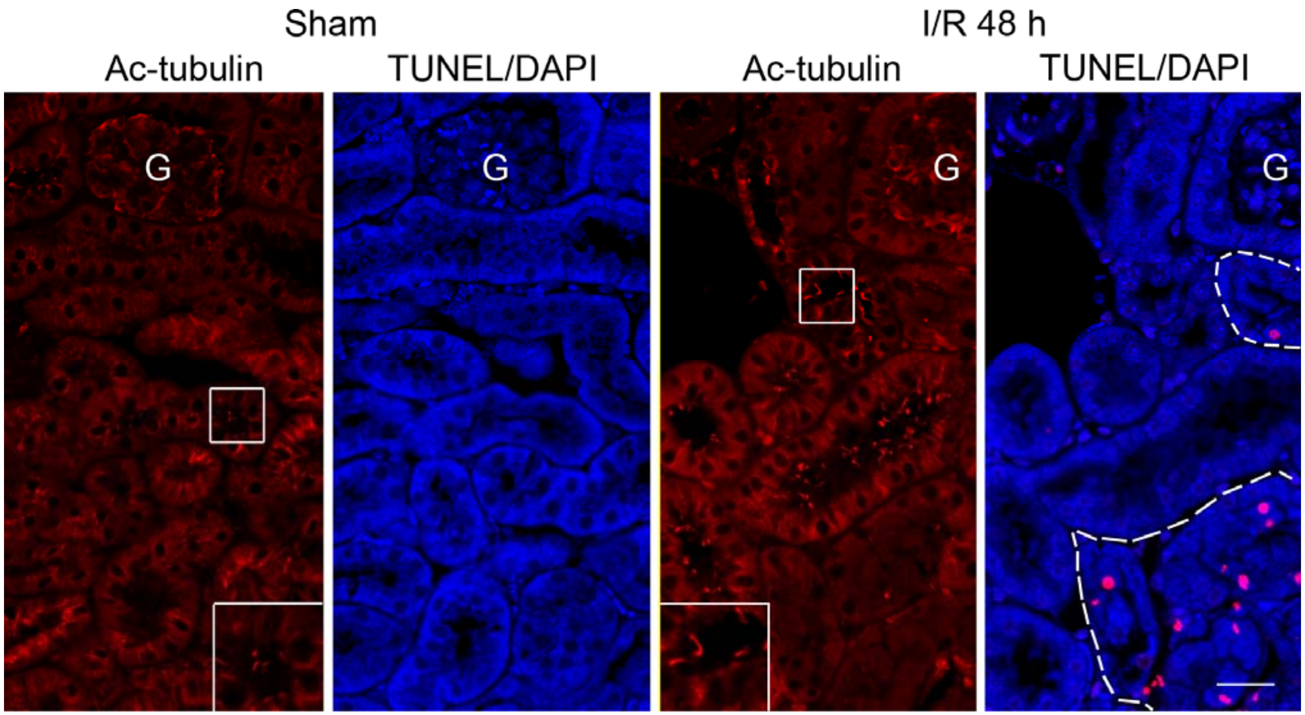

Supplementary Figure 9 Cilium length and cell death in mouse kidney tubules during renal I/R injury. To analyze the association of cilium length and cell death, we performed Ac-tubulin staining and TUNEL assay with kidney sections. After renal I/R, cilium length was increased in TUNEL-negative cells while shortened in TUNEL-positive cells. Please note that Ac-tubulin staining appears weaker in TUNEL-positive cells. Magnified insets are shown at the bottom corner. G, glomerulus. Scale bar, 25 μm.

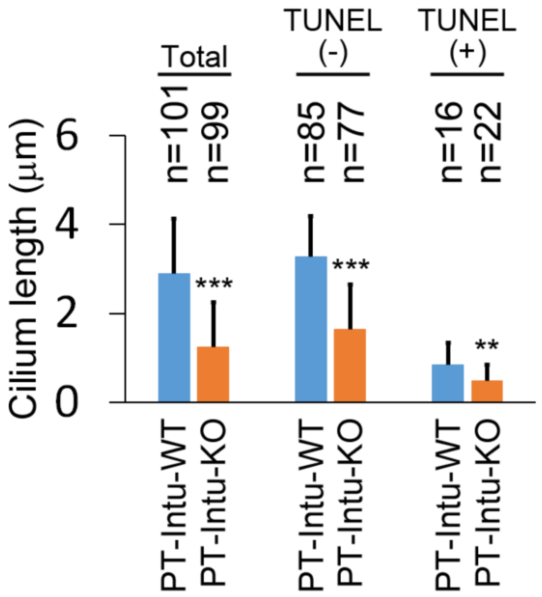

Supplementary Figure 10 Cilium length and cell death in *Intu* KO mice during renal I/R injury. To differentiate the distinction between PT-Intu-WT and –KO mice, we analyzed cilium length in TUNEL-negative and –positive proximal tubular cells. It was observed that cilium length in TUNEL-negative cells was much longer in WT than in KO mice. Similarly, cilium length was shorter in TUNEL-positive KO cells, compared to the TUNEL-positive WT ones. Quantitative data are mean ± s.d. (error bar). Grouped *t* test was used. *p* ** < 0.01, *** <0.001.

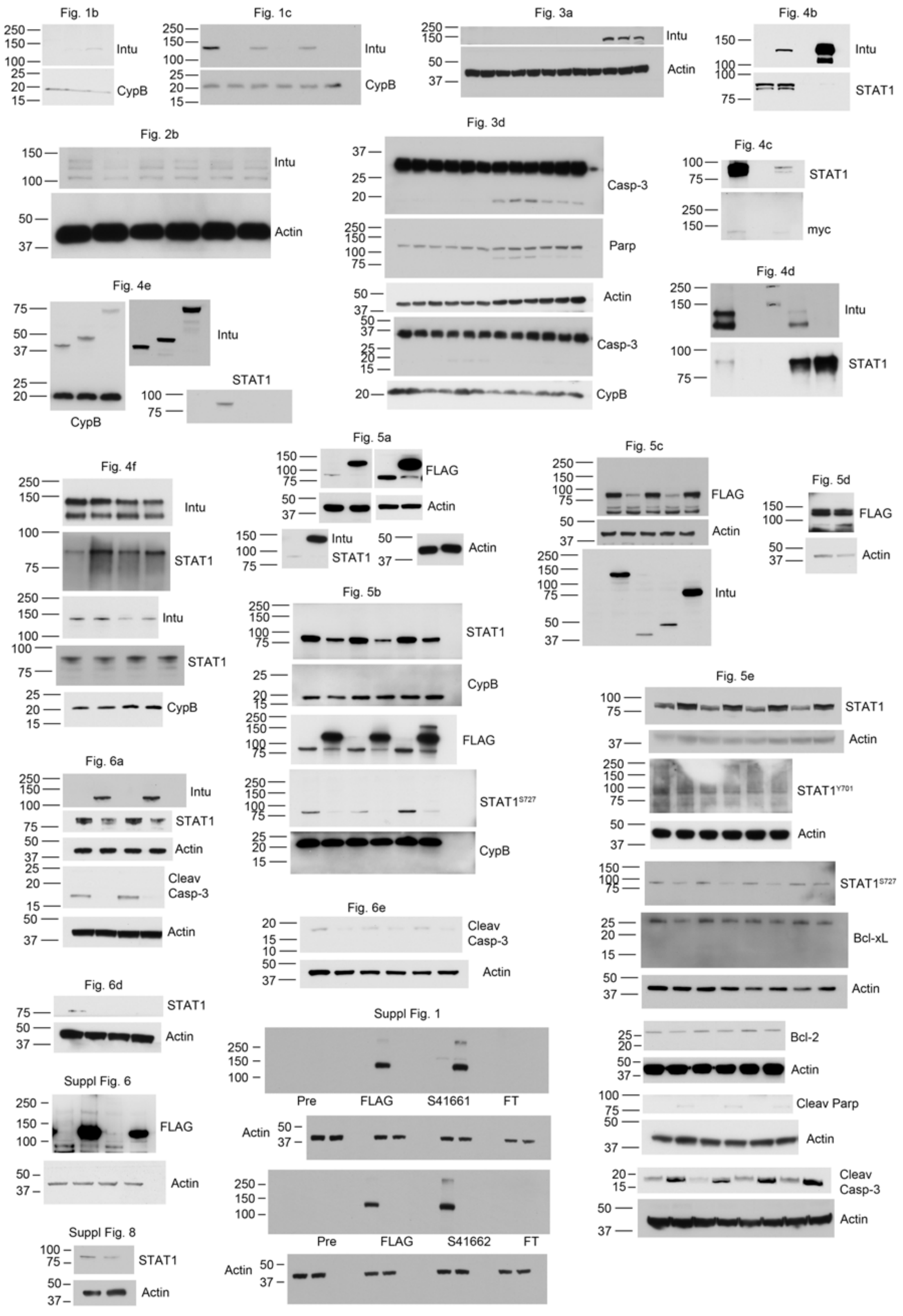

Supplementary Figure 11 Original scans of immunoblots

Supplementary Table 1 Intu-interacting proteins identified in this and previous study

Band ID	Peptide match	This study	Toriyama *et al.* [11]
Band 1	15	Copa	Copa
	13	Sf3b1	Sf3b1
	7	Lmo7	
	4	Myo6	
	3	Vars	Vars
	1	Gigyf2	
	1	Hint1	
	1	Nup160	Nup160
	1	Tbc1d1	
	1	Anln	
Band 2	15	Hsp90ab1	Hsp90ab1
	13	Hsp90aa1	Hsp90aa1
	5	Akap8	Akap8
	4	Dhx15	Dhx15
	3	Mogs	Mogs
	3	Plcd3	Plcd3
	2	Stat1	
	2	Hsp90b1	Hsp90b1
	1	Cpeb4	
	1	Nsun2	Nsun2
Band 3	16	Hspa8	Hspa8
	6	Hspa2	Hspa2
	4	Pcca	
	3	Hspa1a	
	2	Hspa5	Hspa5
	2	Mccc1	Mccc1
	1	Hnrnpm	Hnrnpm
	1	Hspa9	Hspa9
	1	Fip1l1	Fip1l1
Band 4	9	Atp5b	Atp5b
	9	Psmc2	Psmc2
	9	Ifit1	
	7	Hadhb	Hadhb
	7	Ass1	
	6	Poldip3	
	6	Tuba1a	
	6	Eef1g	Eef1g
	5	Ap2m1	Ap2m1
	4	Ddx47	

	4	Atp5a1	Atp5a1
	4	Serpinh1	Serpinh1
	4	Bysl	
	3	Scp2	
	3	Fkbp8	Fkbp8
	3	Tubb2a	Tubb2a
	3	Rbm17	Rbm17
	3	Ighg1	
	2	Krt8	Krt8
	2	Tubb1	
	2	Psmc3	Psmc3
	2	Eef1a1	Eef1a1
	2	Tuba4a	Tuba4a
	2	Tubb5	Tubb5
	2	Psmc4	Psmc4
	2	Rbbp4	Rbbp4
	1	Tubb4a	
	1	Ybx1	Ybx1
	1	Pdia6	Pdia6
	1	Dnaja1	Dnaja1
	1	Cda	
	1	Tubb4b	Tubb4b
	1	Tapbp	
	1	Eif4a1	Eif4a1
	1	Tubb6	Tubb6
	1	Tubg1	
	1	Acat1	
	1	Vim	Vim
	1	Krt7	Krt7
Band 5	6	Acta2	
	3	Uqcrc2	Uqcrc2
	2	Actb	Actb
	1	Mup1	
	1	Eif3g	Eif3g
	1	Erlin1	
	1	Ptrf	Ptrf
	1	Actg1	Actg1
	1	Elmod1	
Band 6	9	Rplp0	Rplp0
	5	Rps3	Rps3
	4	Etfb	

	4	Ldha	
	3	Ywhab	Ywhab
	3	Dhrs4	
	2	Eci1	
	2	Gulp1	
	2	Ywhaz	Ywhaz
	2	Apoa1	
	2	Slc25a10	Slc25a10
	2	Calb1	
	2	Tpi1	Tpi1
	2	Eif3i	Eif3i
	2	Rdh13	Rdh13
	2	Prdx3	
	1	Lrrc59	
	1	Echs1	
	1	Hsd17b10	Hsd17b10
	1	Ywhaq	Ywhaq
	1	Imp4	
	1	Capza1	
	1	Dimt1	
	1	Spr	
	1	Ak2	
	1	Atp5f1	Atp5f1
	1	Try10	
	1	Rps2	
	1	Bphl	
	1	Bdp1	
	1	Ak3	
	1	Psmb6	Psmb6
	1	Inmt	
Band 7	8	Prdx1	
	6	Prdx5	
	5	Bdh2	
	4	Hspb1	Hspb1
	4	Nudt21	
	4	Rpl10a	Rpl10a
	4	Gstz1	
	3	Gstt2	
	3	Arhgdia	
	3	Atp5o	Atp5o
	3	Lypla1	

3	Gsta4	
2	Rab7a	
2	Abhd14b	
2	Prdx6	
2	Prdx2	
2	Gstk1	
2	Ndufs3	Ndufs3
2	Gpx3	
2	Bag2	Bag2
2	Gpx1	
2	Gstm5	
2	Tagln2	
2	Gsta1	
2	Aqp1	
2	Gstm1	
2	Rpl13a	Rpl13a
1	Rab5c	
1	Rab14	Rab14
1	Rab5a	
1	Rab11a	
1	Gstp1	
1	Ndufv2	
1	Rps8	
1	Rpl14	
1	Sod2	
1	Msra	
1	Hprt1	
1	Snap23	
1	Cmpk1	
1	Rab35	
1	Ndufs7	Ndufs7
1	Snrpb	
1	Eif4e2	
1	Rps9	Rps9
1	Eif6	
1	Fam207a	
1	Pafah1b3	
1	Hist1h1e	
1	Cmbl	
